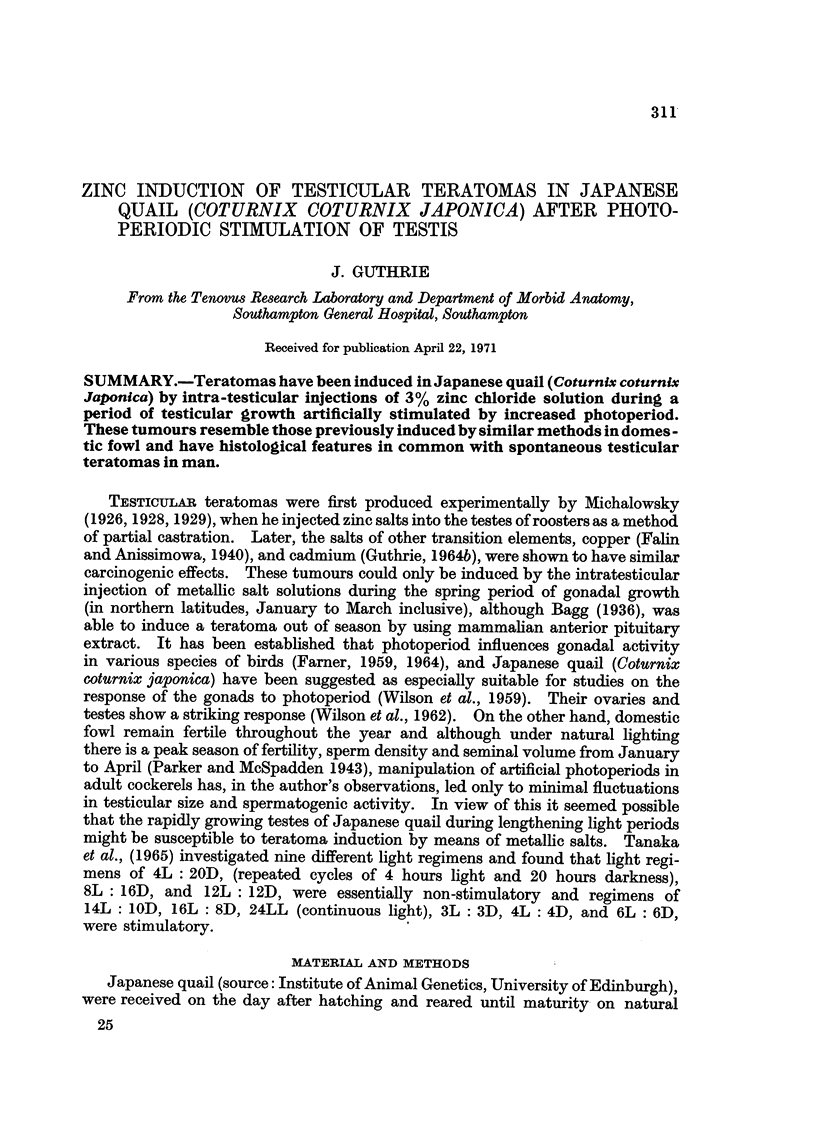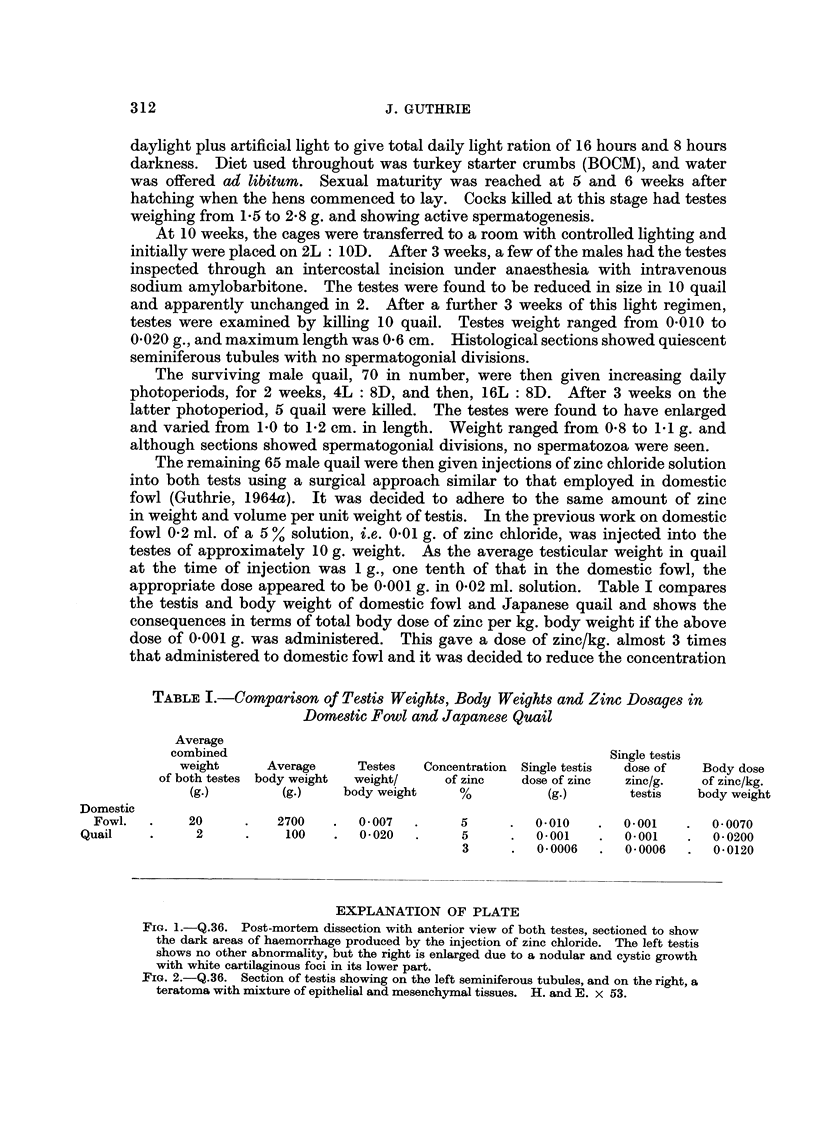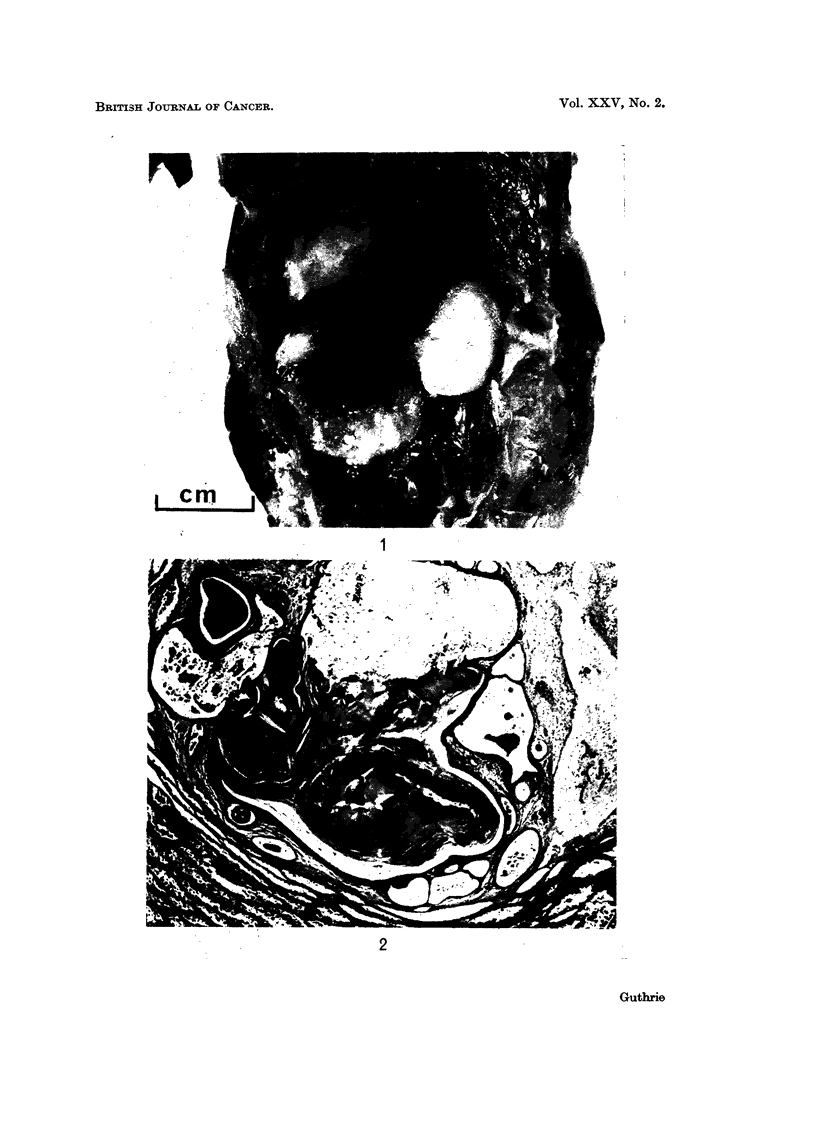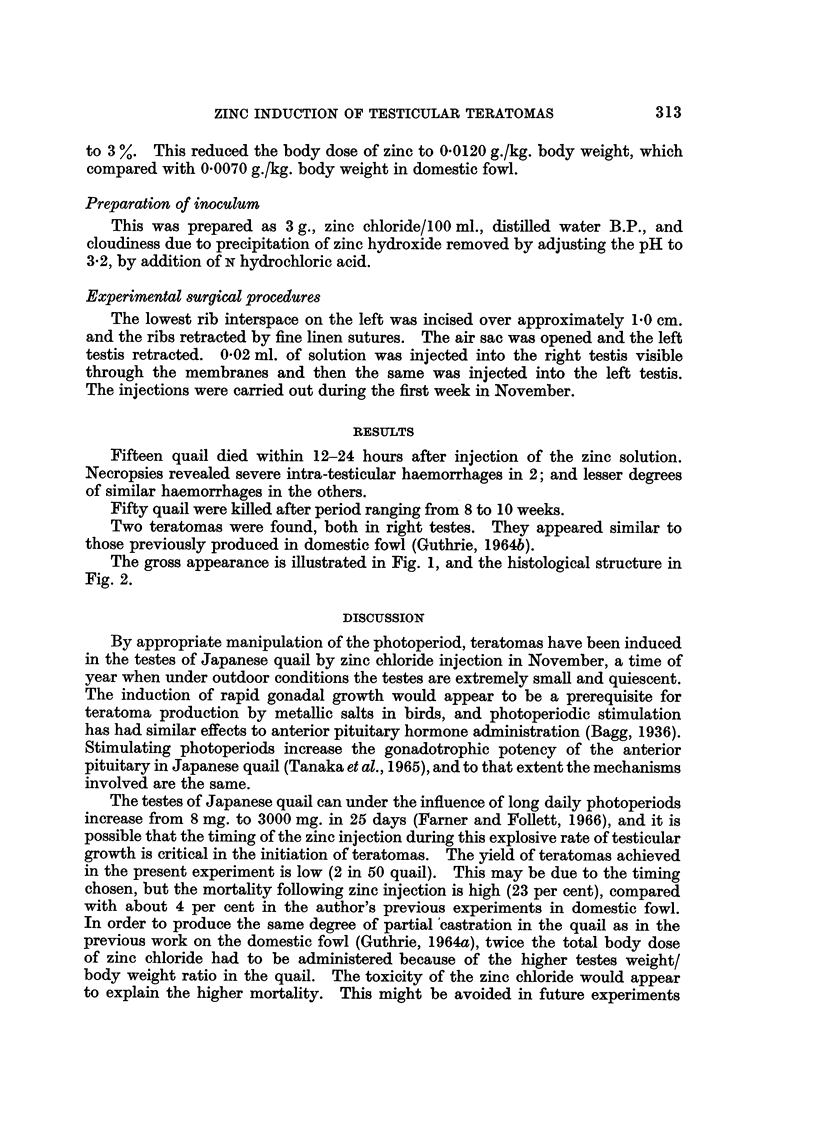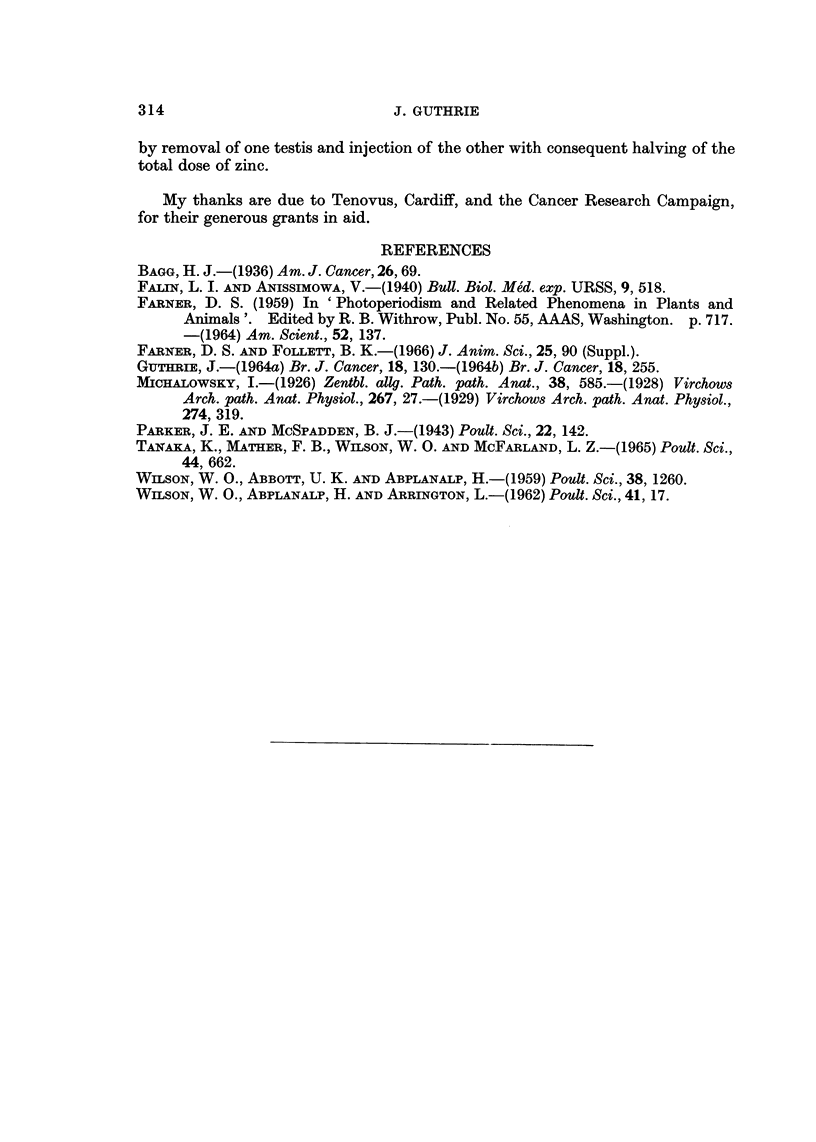# Zinc Induction of Testicular Teratomas in Japanese Quail (Coturnix coturnix japonica) after Photo-periodic Stimulation of Testis

**DOI:** 10.1038/bjc.1971.40

**Published:** 1971-06

**Authors:** J. Guthrie

## Abstract

**Images:**


### ZINC INDUCTION OF TESTICULAR TERATOMAS IN JAPANESE QUAIL (*COTURNIX COTURNIX JAPONICA*) AFTER PHOTOPERIODIC STIMULATION OF TESTIS

J. GUTHRIE

*From the Tenovus Research Laboratory and Department of Morbid Anatomy, Southampton General Hospital, Southampton*

Received for publication April 22, 1971

**SUMMARY.—Teratomas have been induced in Japanese quail (*Coturnix coturnix Japonica*) by intra-testicular injections of 3% zinc chloride solution during a period of testicular growth artificially stimulated by increased photoperiod. These tumours resemble those previously induced by similar methods in domestic fowl and have histological features in common with spontaneous testicular teratomas in man.**

TESTICULAR teratomas were first produced experimentally by Michalowsky (1926, 1928, 1929), when he injected zinc salts into the testes of roosters as a method of partial castration. Later, the salts of other transition elements, copper (Falin and Anissimowa, 1940), and cadmium (Guthrie, 1964*b*), were shown to have similar carcinogenic effects. These tumours could only be induced by the intratesticular injection of metallic salt solutions during the spring period of gonadal growth (in northern latitudes, January to March inclusive), although Bagg (1936), was able to induce a teratoma out of season by using mammalian anterior pituitary extract. It has been established that photoperiod influences gonadal activity in various species of birds (Farner, 1959, 1964), and Japanese quail (*Coturnix coturnix japonica*) have been suggested as especially suitable for studies on the response of the gonads to photoperiod (Wilson *et al.*, 1959). Their ovaries and testes show a striking response (Wilson *et al.*, 1962). On the other hand, domestic fowl remain fertile throughout the year and although under natural lighting there is a peak season of fertility, sperm density and seminal volume from January to April (Parker and McSpadden 1943), manipulation of artificial photoperiods in adult cockerels has, in the author's observations, led only to minimal fluctuations in testicular size and spermatogenic activity. In view of this it seemed possible that the rapidly growing testes of Japanese quail during lengthening light periods might be susceptible to teratoma induction by means of metallic salts. Tanaka *et al.*, (1965) investigated nine different light regimens and found that light regimens of 4L : 20D, (repeated cycles of 4 hours light and 20 hours darkness), 8L : 16D, and 12L : 12D, were essentially non-stimulatory and regimens of 14L : 10D, 16L : 8D, 24LL (continuous light), 3L : 3D, 4L : 4D, and 6L : 6D, were stimulatory.

### MATERIAL AND METHODS

Japanese quail (source: Institute of Animal Genetics, University of Edinburgh), were received on the day after hatching and reared until maturity on natural daylight plus artificial light to give total daily light ration of 16 hours and 8 hours darkness. Diet used throughout was turkey starter crumbs (BOCM), and water was offered *ad libitum.* Sexual maturity was reached at 5 and 6 weeks after hatching when the hens commenced to lay. Cocks killed at this stage had testes weighing from 1·5 to 2·8 g. and showing active spermatogenesis.

At 10 weeks, the cages were transferred to a room with controlled lighting and initially were placed on 2L : 10D. After 3 weeks, a few of the males had the testes inspected through an intercostal incision under anaesthesia with intravenous sodium amylobarbitone. The testes were found to be reduced in size in 10 quail and apparently unchanged in 2. After a further 3 weeks of this light regimen, testes were examined by killing 10 quail. Testes weight ranged from 0·010 to 0·020 g., and maximum length was 0·6 cm. Histological sections showed quiescent seminiferous tubules with no spermatogonial divisions.

The surviving male quail, 70 in number, were then given increasing daily photoperiods, for 2 weeks, 4L : 8D, and then, 16L : 8D. After 3 weeks on the latter photoperiod, 5 quail were killed. The testes were found to have enlarged and varied from 1·0 to 1·2 cm. in length. Weight ranged from 0·8 to 1·1 g. and although sections showed spermatogonial divisions, no spermatozoa were seen.

The remaining 65 male quail were then given injections of zinc chloride solution into both tests using a surgical approach similar to that employed in domestic fowl (Guthrie, 1964*a*). It was decided to adhere to the same amount of zinc in weight and volume per unit weight of testis. In the previous work on domestic fowl 0·2 ml. of a 5% solution, *i.e.* 0·01 g. of zinc chloride, was injected into the testes of approximately 10 g. weight. As the average testicular weight in quail at the time of injection was 1 g., one tenth of that in the domestic fowl, the appropriate dose appeared to be 0·001 g. in 0·02 ml. solution. Table I compares the testis and body weight of domestic fowl and Japanese quail and shows the consequences in terms of total body dose of zinc per kg. body weight if the above dose of 0·001 g. was administered. This gave a dose of zinc/kg. almost 3 times that administered to domestic fowl and it was decided to reduce the concentration

TABLE I.—*Comparison of Testis Weights, Body Weights and Zinc Dosages in Domestic Fowl and Japanese Quail*

| | Average combined weight of both testes (g.) | Average body weight (g.) | Testes weight/ body weight | Concentration of zinc % | Single testis dose of zinc (g.) | Single testis dose of zinc/g. testis | Body dose of zinc/kg. body weight |
|---|---|---|---|---|---|---|---|
| Domestic Fowl. | 20 | 2700 | 0·007 | 5 | 0·010 | 0·001 | 0·0070 |
| Quail | 2 | 100 | 0·020 | 5 | 0·001 | 0·001 | 0·0200 |
| | | | | 3 | 0·0006 | 0·0006 | 0·0120 |

EXPLANATION OF PLATE

FIG. 1.—Q.36. Post-mortem dissection with anterior view of both testes, sectioned to show the dark areas of haemorrhage produced by the injection of zinc chloride. The left testis shows no other abnormality, but the right is enlarged due to a nodular and cystic growth with white cartilaginous foci in its lower part.

FIG. 2.—Q.36. Section of testis showing on the left seminiferous tubules, and on the right, a teratoma with mixture of epithelial and mesenchymal tissues. H. and E. × 53.

1

2

to 3 %. This reduced the body dose of zinc to 0·0120 g./kg. body weight, which compared with 0·0070 g./kg. body weight in domestic fowl.

*Preparation of inoculum*

This was prepared as 3 g., zinc chloride/100 ml., distilled water B.P., and cloudiness due to precipitation of zinc hydroxide removed by adjusting the pH to 3·2, by addition of N hydrochloric acid.

*Experimental surgical procedures*

The lowest rib interspace on the left was incised over approximately 1·0 cm. and the ribs retracted by fine linen sutures. The air sac was opened and the left testis retracted. 0·02 ml. of solution was injected into the right testis visible through the membranes and then the same was injected into the left testis. The injections were carried out during the first week in November.

### RESULTS

Fifteen quail died within 12–24 hours after injection of the zinc solution. Necropsies revealed severe intra-testicular haemorrhages in 2; and lesser degrees of similar haemorrhages in the others.

Fifty quail were killed after period ranging from 8 to 10 weeks.

Two teratomas were found, both in right testes. They appeared similar to those previously produced in domestic fowl (Guthrie, 1964*b*).

The gross appearance is illustrated in Fig. 1, and the histological structure in Fig. 2.

### DISCUSSION

By appropriate manipulation of the photoperiod, teratomas have been induced in the testes of Japanese quail by zinc chloride injection in November, a time of year when under outdoor conditions the testes are extremely small and quiescent. The induction of rapid gonadal growth would appear to be a prerequisite for teratoma production by metallic salts in birds, and photoperiodic stimulation has had similar effects to anterior pituitary hormone administration (Bagg, 1936). Stimulating photoperiods increase the gonadotrophic potency of the anterior pituitary in Japanese quail (Tanaka *et al.*, 1965), and to that extent the mechanisms involved are the same.

The testes of Japanese quail can under the influence of long daily photoperiods increase from 8 mg. to 3000 mg. in 25 days (Farner and Follett, 1966), and it is possible that the timing of the zinc injection during this explosive rate of testicular growth is critical in the initiation of teratomas. The yield of teratomas achieved in the present experiment is low (2 in 50 quail). This may be due to the timing chosen, but the mortality following zinc injection is high (23 per cent), compared with about 4 per cent in the author's previous experiments in domestic fowl. In order to produce the same degree of partial castration in the quail as in the previous work on the domestic fowl (Guthrie, 1964*a*), twice the total body dose of zinc chloride had to be administered because of the higher testes weight/body weight ratio in the quail. The toxicity of the zinc chloride would appear to explain the higher mortality. This might be avoided in future experiments by removal of one testis and injection of the other with consequent halving of the total dose of zinc.

My thanks are due to Tenovus, Cardiff, and the Cancer Research Campaign, for their generous grants in aid.

### REFERENCES

Bagg, H. J.—(1936) *Am. J. Cancer*, **26**, 69.

Falin, L. I. and Anissimowa, V.—(1940) *Bull. Biol. Méd. exp.* URSS, **9**, 518.

Farner, D. S. (1959) In ' Photoperiodism and Related Phenomena in Plants and Animals '. Edited by R. B. Withrow, Publ. No. 55, AAAS, Washington. p. 717. —(1964) *Am. Scient.*, **52**, 137.

Farner, D. S. and Follett, B. K.—(1966) *J. Anim. Sci.*, **25**, 90 (Suppl.).

Guthrie, J.—(1964*a*) *Br. J. Cancer*, **18**, 130.—(1964*b*) *Br. J. Cancer*, **18**, 255.

Michalowsky, I.—(1926) *Zentbl. allg. Path. path. Anat.*, **38**, 585.—(1928) *Virchows Arch. path. Anat. Physiol.*, **267**, 27.—(1929) *Virchows Arch. path. Anat. Physiol.*, **274**, 319.

Parker, J. E. and McSpadden, B. J.—(1943) *Poult. Sci.*, **22**, 142.

Tanaka, K., Mather, F. B., Wilson, W. O. and McFarland, L. Z.—(1965) *Poult. Sci.*, **44**, 662.

Wilson, W. O., Abbott, U. K. and Abplanalp, H.—(1959) *Poult. Sci.*, **38**, 1260.

Wilson, W. O., Abplanalp, H. and Arrington, L.—(1962) *Poult. Sci.*, **41**, 17.